# Bio-Inspired Suture Joints Enable Tuned Load Transfer in Digitally Fabricated Timber with Hybrid Thermoplastic Connectors

**DOI:** 10.3390/biomimetics11100757

**Published:** 2026-10-20

**Authors:** Moisés Sandoval, Carlos Fierro, Geral Moreno, Javier Vidal-Barraza, Juan Pablo Cárdenas-Ramírez, María-José Castilla, Rodrigo Cancino, Rodrigo Valle, César Garrido, Víctor Tuninetti

**Affiliations:** 1 Master Program in Engineering Sciences, Faculty of Engineering and Sciences, Universidad de La Frontera, Temuco 4811230, Chile; msandoval@eagon.cl; 2 R&D Innovation Department, Eagon Lautaro S.A., Ruta 5 Sur Km 644, Lautaro 4860000, Chile; rcancino@eagon.cl; 3 ArGEnCo Department, University of Liège, 4000 Liège, Belgium; 4 Facultad de Arquitectura, Construcción y Medio Ambiente, Universidad Autónoma de Chile, Temuco 4810101, Chile; carlos.fierro1@cloud.uautonoma.cl (C.F.); geral.moreno@cloud.uautonoma.cl (G.M.); javier.vidal@uautonoma.cl (J.V.-B.); 5 Escuela de Construcción Civil, Facultad de Ingeniería, Universidad de Valparaíso, General Cruz 222, Valparaíso 2341266, Chile; juanpablo.cardenas@uv.cl; 6 Faculty of Engineering and Sciences, Universidad de La Frontera, Temuco 4811230, Chile; 7 Construction Multidisciplinary Research Group, Facultad de Arquitectura, Construcción y Medio Ambiente, Universidad Autónoma de Chile, Talca 3460000, Chile; rodrigo.valle@uautonoma.cl; 8 Department of Mechanical Engineering, Universidad del Bío-Bío, Concepción 4081112, Chile; cgarrido@ubiobio.cl; 9 Department of Mechanical Engineering, Universidad de La Frontera, Temuco 4811230, Chile

**Keywords:** digitally fabricated timber, interlocking joint, thermoplastic connector, polypropylene, high-density polyethylene, failure localization

## Abstract

Nature employs complex interlocking geometries, such as vertebrate cranial sutures, to combine load transfer with localized compliance and geometric interlocking. These biological interfaces incorporate alternating wide regions and narrow necks that can accommodate localized deformation while maintaining mechanical interlocking. Inspired by these structural characteristics, digitally fabricated timber systems—such as Skylark–WikiHouse assemblies—can employ analogous bow-tie joint geometries to introduce controlled compliance into the connection. While conventionally manufactured from plywood, the translation of this bio-inspired geometry using hybrid thermoplastic–timber materials remains insufficiently explored. To address this gap, hybrid bow-tie joints comprising 18 mm radiata pine plywood panels and compression-molded polypropylene (PP) or high-density polyethylene (HDPE) connectors were evaluated. Ten joints underwent displacement-controlled monotonic tension at 2 mm/min. The results indicate that PP and HDPE joints achieve nearly identical mean maximum loads of 6.61 and 6.65 kN, respectively. However, substituting PP with HDPE significantly alters the global load–displacement response, yielding 33% lower global stiffness, 63% greater displacement at maximum load, and 70% greater external mechanical work. Post-test examinations revealed that final visible cracking was localized to the surrounding plywood, with no complete rupture of the thermoplastic connectors. This final damage localization is consistent with the intended suture-inspired design concept; however, the present post-test observations do not establish the temporal sequence of crack initiation and propagation or directly demonstrate crack arrest. These findings identify connector material as a practical bio-inspired design variable for tuning the global stiffness–deformation response of digitally fabricated timber joints while maintaining comparable maximum tensile load capacity.

## 1. Introduction

Nature has evolved load-bearing protective systems in which stiff components are joined by compliant sutural interfaces, combining mechanical protection with localized deformation [1,2]. Cranial and turtle-shell sutures can be modeled as interlocking bone–suture–bone systems, where morphological complexity and the mechanical properties of the softer interface influence stress distribution, deformation, and strain-energy absorption [1,2,3]. Studies of suture-inspired profiles further show that contact angle, tangent length, tab radius, contact area, segmentation, and loading position influence stiffness, toughness, and strain-energy localization [1,2,3,4]. These observations motivate the use of controlled local compliance and geometric interlocking as design principles for engineered interfaces, without implying that a macroscopic structural analog necessarily reproduces the underlying biological damage mechanisms. Similar biomimetic design strategies have increasingly been investigated in architected materials, where geometry, deformation control, and energy absorption are combined to tailor mechanical performance across different length scales [5].

These biomechanical principles have informed the growing use of structural interlocking in architectural and mechanical engineering. Digital fabrication links computational design to automated manufacturing, allowing construction components to be produced directly from digital models and enabling fabrication-aware structural design [6,7,8]. In timber construction, WikiHouse applies this approach through CNC machining of sheet-based components that assemble through digitally defined interlocking connections [9,10,11,12,13]. This approach is also attractive because engineered timber and reversible construction can reduce embodied emissions, prolong biogenic-carbon storage, and facilitate disassembly and material reuse, although these outcomes depend on sourcing, processing, service life, and end-of-life assumptions [14,15,16,17,18].

For these systems, structural behavior is governed not only by the timber members but also by the joints that transfer load between them [19,20]. The mechanical response of the plywood substrate is itself direction-dependent and heterogeneous, with recent experimental characterization reporting distinct in-plane and through-thickness shear responses associated with the orthotropic structure of the panel [21]. Interlocking wood connections transmit load primarily through contact, bearing, and friction at mating surfaces; depending on the system, they can reduce reliance on conventional steel fasteners or adhesives [22,23,24,25]. Their response is consequently sensitive to connector geometry, dimensional tolerances, fabrication accuracy, and the distribution of local contact forces [26,27,28,29]. Because these interfacial contributions can evolve with deformation and differ between material pairs, changes in connector material may affect the measured global response through both bulk compliance and interface-level interactions. These contributions therefore need to be distinguished from material stiffness alone when interpreting experimental joint-level measurements. Joint-level testing is therefore essential when evaluating a new connection concept, because connector type and arrangement influence stiffness, load-carrying capacity, displacement response, and failure mode [30,31,32].

WikiHouse-style assemblies provide a useful setting in which to translate suture-inspired design principles into timber construction. Their bow-tie or dovetail-type connectors resist separation through geometric interlocking while retaining the potential for localized relative movement under load. However, these connectors are commonly manufactured from rigid plywood. Whether their load-transfer path, global mechanical response and final damage localization can be deliberately tuned by introducing a compliant connector material, such as a thermoplastic, remains insufficiently explored.

Research on digitally fabricated and carpentry-style timber connections has focused predominantly on geometric development, fabrication constraints, and the mechanical response of all-wood joints [20,33,34,35]. Studies of interlocking systems in other material classes similarly show that geometry and orientation influence tensile resistance and failure development in compliant, composite, and additively manufactured joints [36,37,38,39]. These findings establish geometry as a governing design variable. Nevertheless, they provide limited evidence regarding the extent to which the global mechanical response and final damage localization of a digitally fabricated timber joint can be modified through connector-material substitution while retaining the connection geometry.

Hybrid timber connections have been investigated using timber–concrete systems and combinations of mechanical and bonded joining mechanisms [40,41]. More broadly, hybrid joining combines different materials or joining techniques to obtain joint properties that are not necessarily achieved by either constituent approach alone [42]. Related work on architected polymeric metamaterials has likewise shown that combining a designed cellular geometry with a polymeric matrix can modify stiffness and energy-absorption characteristics, demonstrating how material selection can complement geometric design in tailoring mechanical response [43]. Recent research has also demonstrated the feasibility of joining wood and thermoplastic components through ultrasonic processing [44]. However, much of the available wood–polymer literature concerns bonded interfaces, wood-based polymer composites, or additive-manufacturing feedstocks rather than a discrete molded thermoplastic insert acting as the primary load-transfer component of an interlocking timber joint [44,45,46]. The mechanical response of such an insert cannot be inferred directly from bonded or composite systems because load transfer in the present configuration occurs predominantly through geometric interlocking and surface contact. Accordingly, nominal bulk polymer properties provide a useful material reference but cannot by themselves characterize the joint-level response of the molded connector within the assembled timber interface.

Compression molding provides a non-layerwise route for manufacturing thermoplastic components with a prescribed geometry. Processing conditions can influence molecular orientation and the resulting mechanical response of compression-molded high-density polyethylene (HDPE) components [47]. Previous research has also evaluated the structural performance of HDPE and wood–plastic composite lumber components in load-bearing applications, demonstrating the importance of component-level characterization when polymeric materials perform a structural function [48]. This consideration is particularly relevant when nominal resin properties are used to interpret the response of a molded structural connector, because the properties of the finished component may depend on processing history, geometry, and manufacturing conditions. Polypropylene (PP) and HDPE were therefore selected in the present study as two thermoplastics with different nominal stiffnesses and deformation characteristics, allowing for the connector material to be examined while the bow-tie geometry, plywood configuration, and loading protocol were maintained.

Accordingly, this study investigates the monotonic tensile response and final visible damage localization of hybrid Skylark–WikiHouse bow-tie joints incorporating compression-molded PP and HDPE connectors. The specific objectives are to determine the influence of connector material on (i) maximum tensile load, (ii) crosshead displacement at maximum load, (iii) global joint stiffness evaluated between 10% and 40% of maximum load, (iv) external mechanical work up to maximum load, and (v) the final visible crack pattern and the occurrence of connector rupture. Based on the nominal contrast between the two thermoplastics, HDPE joints are expected to exhibit lower global stiffness and greater displacement and external mechanical work up to maximum load than PP joints, while reaching maximum loads of a similar order. Previously reported all-plywood Skylark–WikiHouse TIE joints are used only as contextual benchmarks because those results were obtained in a separate experimental campaign [20]. By maintaining a fixed connector geometry and plywood configuration, the study examines whether thermoplastic selection can modify the global stiffness–deformation response without changing the connection topology. The experimental design is intended to establish differences in the measured global joint response and final post-test damage state; it does not directly resolve local strain fields, interfacial friction, crack-initiation timing, or the constitutive response of the molded thermoplastic connectors.

## 2. Materials and Methods

### 2.1. Constituent Materials

Structural *Pinus radiata* plywood with a nominal thickness of 18 mm was used for all timber components. The plywood was supplied by EAGON Lautaro S.A., Lautaro, Chile and consisted of seven cross-laminated veneers. The outer veneers were oriented parallel to the tensile loading direction in all specimens. Before specimen fabrication, the plywood panels were conditioned under laboratory conditions at 20±2 °C until a moisture content of 10.5±0.5% was reached. According to the manufacturer’s technical specifications, the plywood density ranged from 0.48 to 0.55 g/cm^3^. Commercial-grade polypropylene (PP), grade R520Y (SK Energy Co., Ltd., Seoul, Republic of Korea), and commercial-grade high-density polyethylene (HDPE), grade 2520 (SK geo centric Co., Ltd., Seoul, Republic of Korea), were used to manufacture the thermoplastic bow-tie connectors. Before compression molding, the polymer feedstocks were cleaned, mechanically ground, and pelletized to obtain homogeneous feedstock suitable for compression molding. Table 1 summarizes the identification and reference properties of the constituent materials. The plywood properties were obtained from a previous orthotropic characterization of the same 18 mm *Pinus radiata* plywood batch used in the present experimental program [49]. The PP and HDPE properties were obtained from the corresponding manufacturer datasheets. These values characterize the commercial resin grades and were not measured directly from the compression-molded connectors examined in this study.

The manufacturer-datasheet tensile strengths of PP and HDPE are of a similar order, whereas HDPE has a lower nominal tensile modulus than PP. This contrast provides the material basis for examining whether connector material modifies the global response of joints manufactured with the same bow-tie geometry. Because the reported thermoplastic properties correspond to the commercial resin grades rather than to tensile specimens extracted from the molded connectors, they are treated only as nominal reference characteristics. They are not used as specimen-specific constitutive properties or as direct measurements of the mechanical response of the finished connectors. Differences between the nominal resin properties and the properties of the molded components may arise from processing history, component geometry, and manufacturing variability.

### 2.2. Fabrication of Bio-Inspired Thermoplastic Connectors

**Figure 1:**
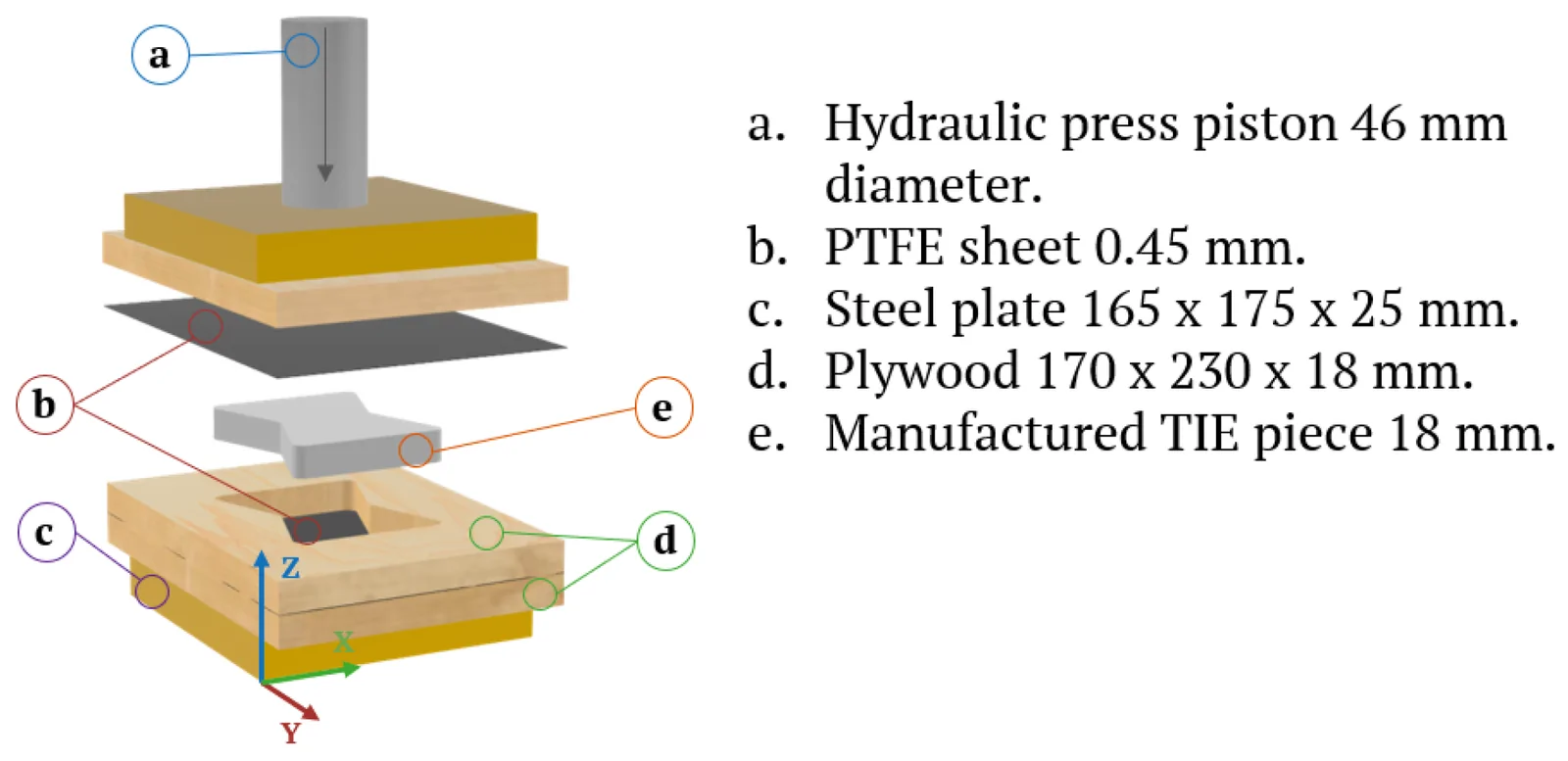
Compression-molding procedure used to manufacture the thermoplastic bow-tie connectors, including feedstock heating and melting, transfer of the molten material to the mold cavity, and forming under compressive load.

Figure 1 presents the compression-molding procedure used to manufacture the thermoplastic connectors. The mold replicated the geometry of the bow-tie connector used in the Skylark–WikiHouse TIE joint. The PP feedstock was heated at 200 °C for 10 min, whereas the HDPE feedstock was heated at 180 °C for 15 min. The molten material was then transferred immediately to the mold cavity and compressed under a load of 49 kN for 10 min. The use of material-specific processing conditions is relevant because variations in thermal processing parameters can influence the resulting thermomechanical response of polymeric materials [50]. The different heating conditions used for PP and HDPE were part of the respective processing procedures and were not independently varied as experimental factors. Consequently, the present comparison evaluates the molded connector materials as manufactured under these conditions, rather than isolating the effect of polymer chemistry from processing history.

After molding, the connectors were allowed to cool to room temperature for at least 24 h before demolding. Excess material generated during molding was removed by mechanical trimming. Before assembly, each connector was visually examined for incomplete cavity filling, visible surface voids, incomplete fusion, and surface cracking. The inspection was qualitative; internal void content and dimensional deviations were not quantified.

### 2.3. Bio-Inspired Specimen Geometry and Tensile Testing

**Figure 2:**
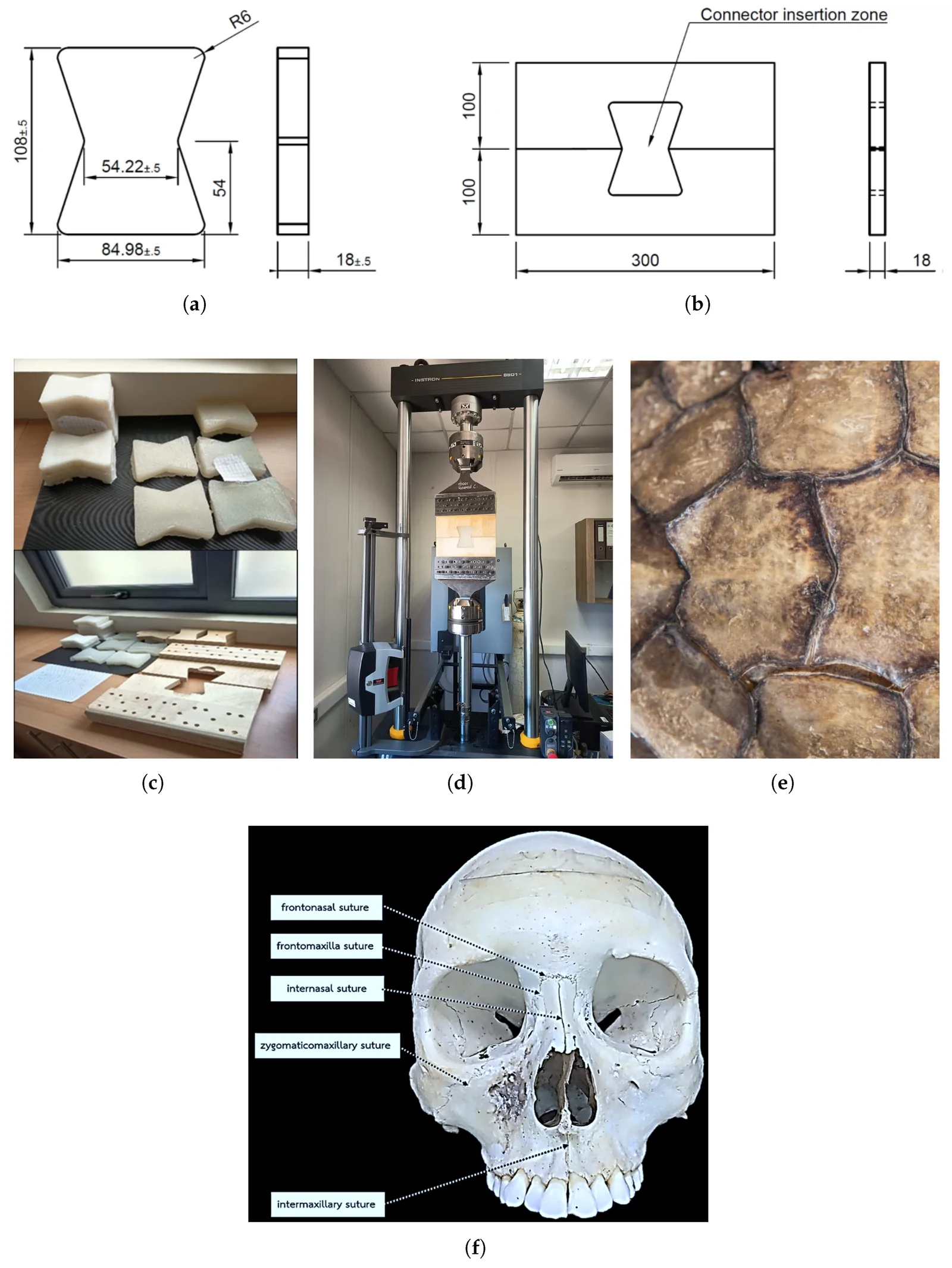
Bio-inspired specimen geometry, manufactured hybrid components, and monotonic tensile-test configuration: (**a**) structural bow-tie connector dimensions designed to emulate natural re-entrant interlocking mechanisms; (**b**) rigid plywood-panel geometry and connector insertion region; (**c**) manufactured compliant thermoplastic interfaces (PP and HDPE) alongside plywood panels, mirroring biological bone–suture–bone assemblies; (**d**) assembled hybrid specimen installed in the Instron 8801 testing machine; (**e**) reference biological morphology of a turtle carapace demonstrating the inspiration for interdigitated load-transfer joints; and (**f**) human facial sutures illustrating natural re-entrant interlocking mechanisms (adapted from [51] under CC-BY 4.0 license). Dimensions for (**a**,**b**) are given in millimeters.

Figure 2 presents the specimen geometry, manufactured components, and tensile-test configuration. Each joint consisted of two 18 mm thick plywood panels connected by one molded thermoplastic bow-tie insert. The connector and plywood-panel dimensions are shown in Figure 2a and Figure 2b, respectively. The manufactured components and assembled tensile-test configuration are shown in Figure 2c,d.

Ten hybrid joints were tested, comprising five PP specimens (P1–P5) and five HDPE specimens (P6–P10). The tests were conducted using an Instron 8801 servo-hydraulic testing machine (Instron, Norwood, MA, USA) equipped with a 100 kN load cell. The specimens were clamped using custom grips manufactured from ASTM A36 steel. The grips incorporated tapered loading plates whose thickness decreased from 10 to 6 mm to promote stable load transfer and limit loading eccentricity.

All assembled specimens were stored under the same laboratory conditions. Monotonic tensile loading was applied under displacement control at a constant crosshead rate of 2 mm/min. Each test was terminated when the post-peak force decreased by more than 40% of the maximum tensile load, corresponding to a recorded force below 60% of $F_{\max}$, or when the crosshead displacement reached the safety limit of 30 mm, whichever occurred first.

No extensometer or full-field displacement measurement was used. Consequently, all displacement values reported in this study were obtained from the testing-machine crosshead and represent the global response of the specimen–grip–machine system rather than local deformation of the thermoplastic connector or plywood. The measured crosshead displacement therefore includes contributions from deformation of the joint components, relative movement at the interlocking contact surfaces, grip compliance, and testing-machine compliance. The present experimental configuration does not permit these contributions to be separated independently. Accordingly, $\delta_{\max}$ and $k_{10-40}$ are interpreted as global joint-level response parameters rather than direct measures of connector deformation or intrinsic connector stiffness.

The interlocking load-transfer mechanism involves contact and bearing at the mating surfaces and may also involve friction. The present study did not independently characterize the coefficient of friction, contact-pressure distribution, contact-area evolution, or interface slip for the PP–plywood and HDPE–plywood pairs. These interfacial effects are therefore considered possible contributors to the measured global response but are not quantitatively separated from bulk connector compliance in the present analysis.

### 2.4. Response Parameters and Data Reduction

The force–displacement response of each joint was recorded continuously throughout testing. Four scalar response parameters were extracted: maximum tensile load, $F_{\max}$; crosshead displacement at maximum load, $\delta_{\max}$; global joint stiffness, $k_{10-40}$; and external mechanical work up to maximum load, $W_{\mathrm{pre}}$. The global joint stiffness was evaluated over the approximately linear portion of the ascending force–displacement response between 10% and 40% of $F_{\max}$. For each specimen, the data within this interval were fitted by linear regression according to

(1) $$F_{i}=\beta_{0}+k_{10-40}\delta_{i}+\varepsilon_{i},$$

where $F_{i}$ and $\delta_{i}$ are the recorded force and crosshead displacement values, respectively; $\beta_{0}$ is the fitted intercept; $k_{10-40}$ is the regression slope; and $\varepsilon_{i}$ is the residual term. Because $\delta_{i}$ is obtained from the testing-machine crosshead, $k_{10-40}$ represents the stiffness of the complete specimen–grip–machine response over the specified load interval and is not interpreted as an intrinsic stiffness of the thermoplastic connector. The resulting slope is reported as global joint stiffness because the crosshead displacement includes compliance from the specimen, grips, and testing machine. The external mechanical work accumulated up to maximum load was calculated as the area under the force–displacement curve from the beginning of loading to $\delta_{\max}$:

(2) $$W_{\mathrm{pre}}=10^{-3}\int_{0}^{\delta_{\max}}F\left(\delta \right)\;d\delta ,$$

where *F* is expressed in newtons, δ in millimeters, and $W_{\mathrm{pre}}$ in joules. The integral was evaluated numerically from the recorded force–displacement data using the trapezoidal rule. Because the calculated area was initially obtained in N mm, it was multiplied by $10^{-3}$ to convert the result to joules. The parameter $W_{\mathrm{pre}}$ represents the external mechanical work associated with the measured global response up to maximum load. It is not interpreted as fracture energy, intrinsic material toughness, or energy dissipated exclusively by the thermoplastic connector. Because the displacement coordinate is based on the testing-machine crosshead, $W_{\mathrm{pre}}$ represents the external work applied to the complete specimen–grip–machine system up to maximum load and cannot be partitioned into connector, plywood, interface, grip, and machine contributions using the present measurements.

Post-test photographs of the front and rear specimen surfaces were used to document the final visible surface crack trajectories, visible deformation of the connector, and the presence or absence of connector rupture. Because continuous optical or full-field measurements were not performed, the post-test images were not used to determine crack-initiation time or the complete temporal sequence of crack propagation.

### 2.5. Descriptive and Comparative Analysis

For each connector material (n=5), the measured response parameters were summarized using the arithmetic mean, sample standard deviation, and coefficient of variation. The coefficient of variation was calculated as

(3) $$\mathrm{CV}=100\frac{s}{\bar{x}},$$

where *s* is the sample standard deviation and $\bar{x}$ is the arithmetic mean. Comparisons between the PP and HDPE groups were treated as descriptive comparisons of the measured responses. No statistical hypothesis test was used to infer population-level differences between the two connector materials. Previously published results for all-plywood Skylark–WikiHouse TIE joints [20] were used only as contextual benchmarks because they originated from a separate experimental campaign and comprised two specimens per plywood orientation. These historical data were therefore not treated as a concurrent control group or included in inferential comparisons.

## 3. Results and Discussion

### 3.1. Load Transfer and Global Tensile Response

Table 2 presents the maximum tensile load, $F_{\max}$, and crosshead displacement at maximum load, $\delta_{\max}$, measured for the PP and HDPE joints. The PP specimens reach maximum loads between 5.79 and 7.49 kN, whereas the HDPE specimens reached values between 6.04 and 7.16 kN. The corresponding group means were 6.61 and 6.65 kN, representing a difference of approximately 0.7%. The dispersion in maximum load was lower for HDPE, with coefficients of variation of 7.2% for HDPE and 10.6% for PP.

The principal distinction between the two connector groups is observed in their displacement response. The mean crosshead displacement at maximum load increased from 9.31 mm for PP to 15.16 mm for HDPE, corresponding to an increase of approximately 63%. The HDPE group also exhibited greater specimen-to-specimen variability in $\delta_{\max}$, with a coefficient of variation of 29.1%, compared with 16.2% for PP. Specimen P9 reached the largest displacement at maximum load, 22.52 mm, while attaining a maximum load of 7.16 kN.

**Figure 3:**
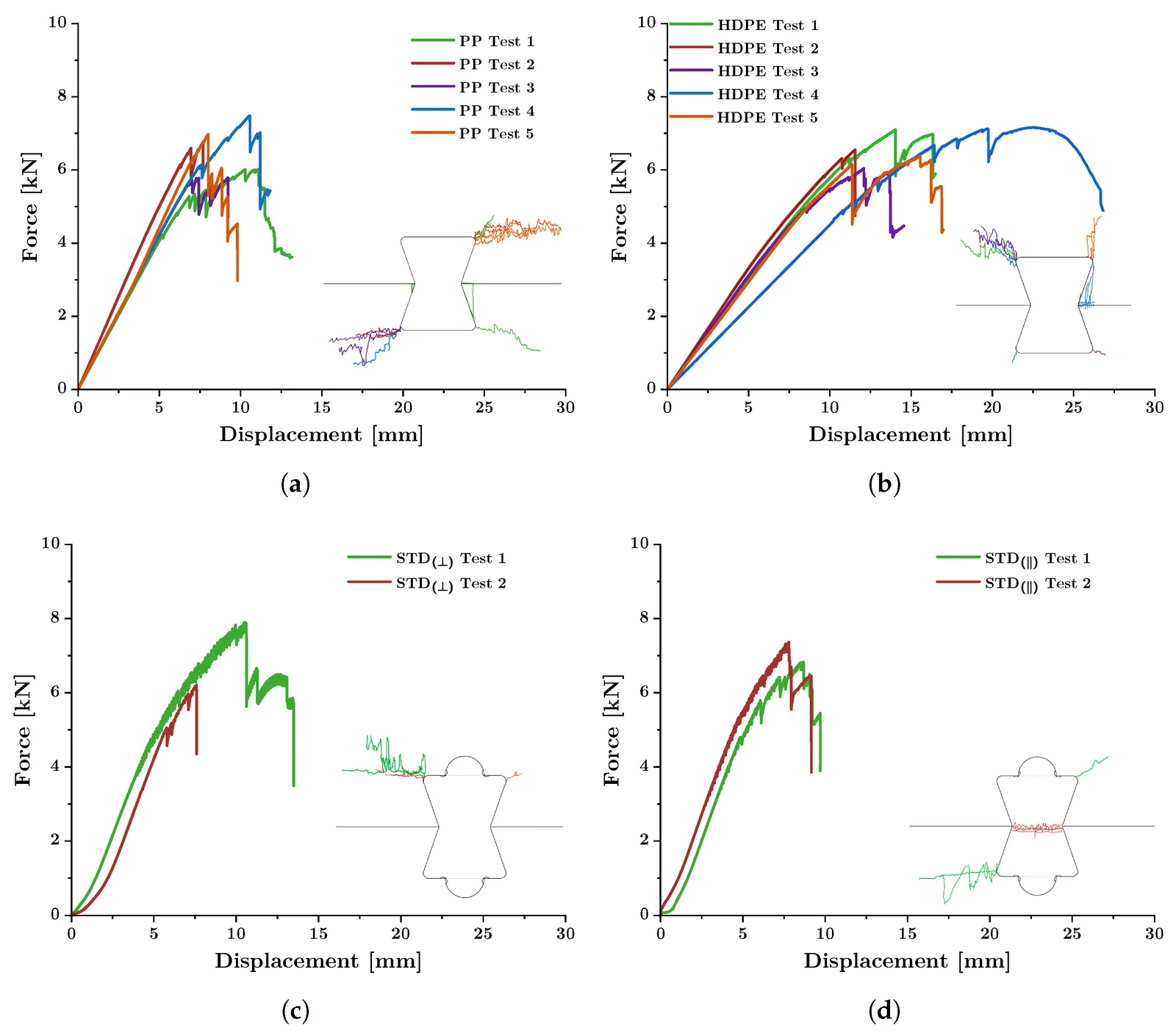
Force–crosshead-displacement responses of Skylark–WikiHouse bow-tie joints subjected to monotonic tensile loading: (**a**) PP connectors; (**b**) HDPE connectors; (**c**) previously reported all-plywood joints with the perpendicular configuration, STD_(⊥)_; and (**d**) previously reported all-plywood joints with the parallel configuration, STD_(‖)_. The all-plywood results were obtained in a separate experimental campaign and are included as contextual benchmarks from Sandoval et al. [20].

Figure 3 presents the measured force–crosshead-displacement curves for the PP and HDPE joints, together with previously reported curves for the all-plywood Skylark–WikiHouse configurations [20]. The latter are included only as contextual benchmarks because they were obtained in a separate experimental campaign with two specimens per plywood orientation.

The PP and HDPE curves generally exhibit an approximately linear ascending response at low displacement, followed by a progressive departure from linearity. Several curves contain intermittent force reductions before or after maximum load. The global records alone do not establish the physical origin of each force reduction because local strain fields and the temporal development of cracking were not measured. Nevertheless, the curves clearly show that the HDPE joints sustain larger crosshead displacements before maximum load than the PP joints, while the ranges of maximum load overlap substantially.

The contextual all-plywood curves reach mean maximum loads of approximately 7.10 kN for the parallel configuration and 7.05 kN for the perpendicular configuration [20]. These values are of the same general order as the present PP and HDPE results. However, the historical curves extend over a shorter displacement range and exhibit a steeper initial response. Because the plywood results were obtained separately, these observations are descriptive and do not constitute evidence of statistical equivalence, superiority, or non-inferiority among the four configurations.

### 3.2. Tunable Compliance: Joint Stiffness and External Mechanical Work

Table 3 summarizes the four global response parameters considered in this study. The PP joints exhibit a mean global joint stiffness, $k_{10-40}$, of 875.9±79.3 N/mm, whereas the HDPE joints exhibit a mean value of 587.4±81.8 N/mm. The mean stiffness of the HDPE group is therefore approximately 33% lower than that of the PP group.

The opposite trend is observed for external mechanical work up to maximum load, $W_{\mathrm{pre}}$. The mean value increased from 35.2±7.6 J for PP to 59.7±22.7 J for HDPE, corresponding to an increase of approximately 70%. The greater $W_{\mathrm{pre}}$ measured for the HDPE joints results from the combination of maximum loads of the same general magnitude and the larger global displacement accumulated before maximum load. Because $W_{\mathrm{pre}}$ was calculated using force and crosshead displacement, it characterizes the external mechanical work associated with the complete specimen–grip–machine response up to maximum load. It does not represent fracture energy, intrinsic material toughness, or energy dissipated exclusively through deformation of the thermoplastic connector.

The measured PP–HDPE contrast is consistent with a contribution from the nominal stiffness difference reported for the two commercial thermoplastic grades. HDPE has a lower manufacturer-datasheet tensile modulus than PP, which is consistent with the lower $k_{10-40}$ and greater $\delta_{\max}$ measured for the HDPE joints. However, this correspondence does not establish elastic modulus as the sole cause of the observed group differences. The mechanical response may also be affected by other material characteristics, compression-molding history, local fit, dimensional variability, and interfacial contact conditions, none of which was isolated independently in the present experiment.

Because the interlocking load-transfer mechanism involves contact, bearing, and friction, differences in the PP–plywood and HDPE–plywood interfaces may also contribute to the measured global response. The coefficient of friction, contact-pressure distribution, contact-area evolution, and possible interface slip were not independently characterized in the present study. These effects therefore remain possible contributors to the observed differences but cannot be quantitatively separated from the bulk compliance of the joint using the present measurements.

**Figure 4:**
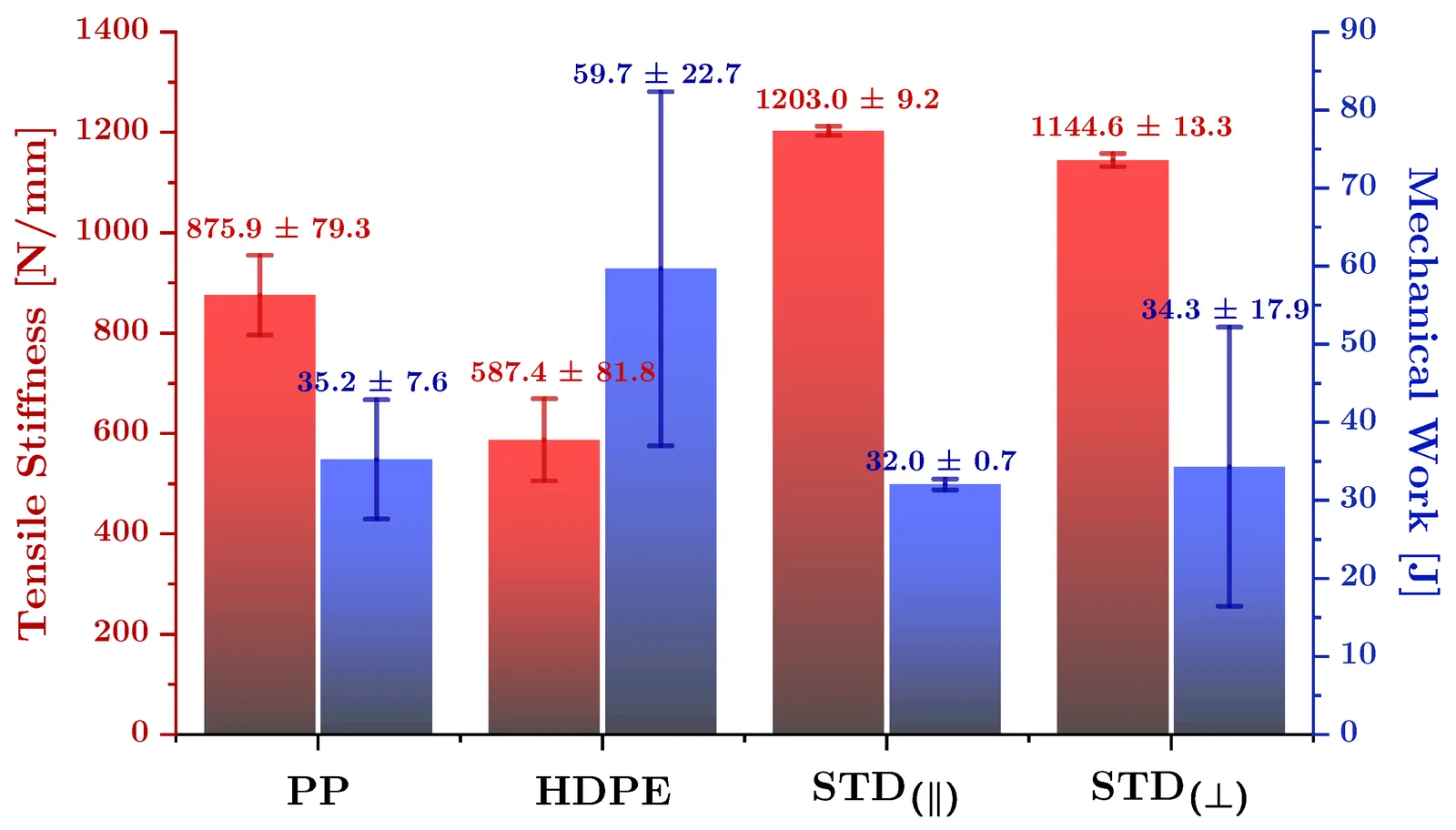
Comparison of global joint stiffness, $k_{10-40}$, and external mechanical work up to maximum load, $W_{\mathrm{pre}}$, for the PP and HDPE joints and the previously reported all-plywood configurations. Values are shown as mean ± standard deviation. The plywood results are contextual benchmarks from Sandoval et al. [20].

Figure 4 provides a graphical comparison of $k_{10-40}$ and $W_{\mathrm{pre}}$. The previously reported all-plywood joints exhibit higher mean global stiffness than either thermoplastic group. Relative to PP, the historical parallel and perpendicular configurations are approximately 37% and 31% stiffer, respectively. Their mean external-work values, however, are close to that of the PP group: 32.0 and 34.3 J for the parallel and perpendicular plywood configurations, compared with 35.2 J for PP. The mean external mechanical work of the HDPE group is approximately 87% greater than that of the historical parallel configuration and 74% greater than that of the historical perpendicular configuration. These percentages provide engineering context but should not be interpreted inferentially because the historical datasets contain only two specimens per orientation and were obtained in a separate campaign.

The combined response parameters identify a clear contrast between the two thermoplastic groups. PP produces the stiffer global response and reaches maximum load at a lower mean displacement, whereas HDPE permits greater global displacement and external mechanical work before maximum load. This contrast is substantially greater than the difference in mean maximum load, which remains below 1% between the two groups.

### 3.3. Damage Localization and Bio-Inspired Interpretation

**Figure 5:**
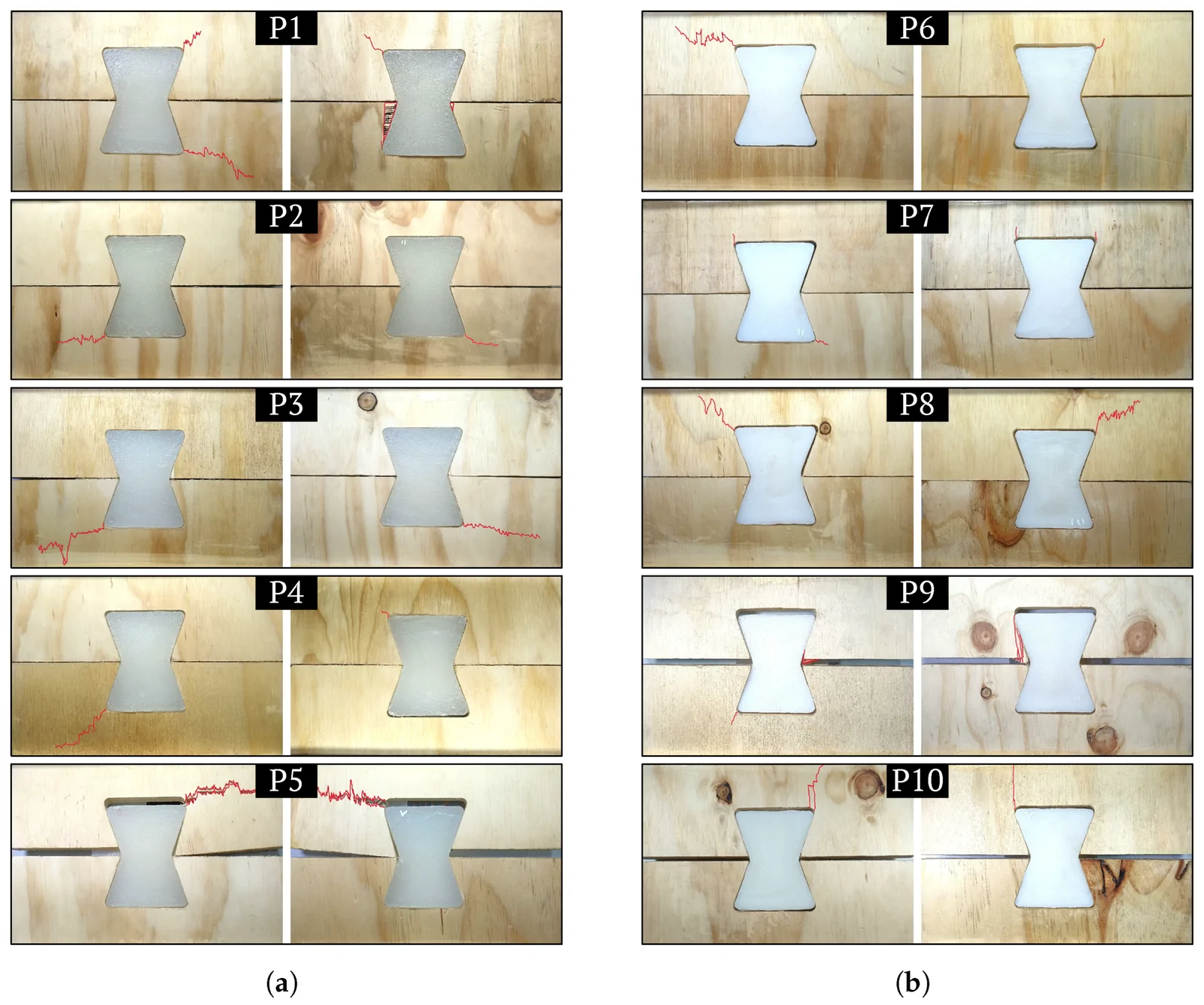
Post-test condition of the hybrid bow-tie joints: (**a**) PP specimens P1–P5 and (**b**) HDPE specimens P6–P10. The images document the final visible condition of the specimens after monotonic tensile testing, with red lines indicating the crack propagation paths.

Figure 5 presents the post-test condition of the PP and HDPE specimens. Final visible cracking was located in the surrounding plywood in all ten hybrid specimens. No complete rupture of either thermoplastic connector type was observed before the applicable test-termination criterion was reached.

The PP connectors exhibit comparatively limited visible geometric change after testing. In contrast, several HDPE connectors exhibit visible permanent elongation and localized narrowing while remaining unruptured. These observations document differences in the final visible condition of the connectors, but they do not provide a quantitative measure of local connector strain or plastic deformation. Because the displacement measurements were obtained from the testing-machine crosshead, the additional global displacement measured for HDPE cannot be partitioned among connector deformation, plywood deformation, relative movement at the contact surfaces, grip compliance, and testing-machine compliance.

**Figure 6:**
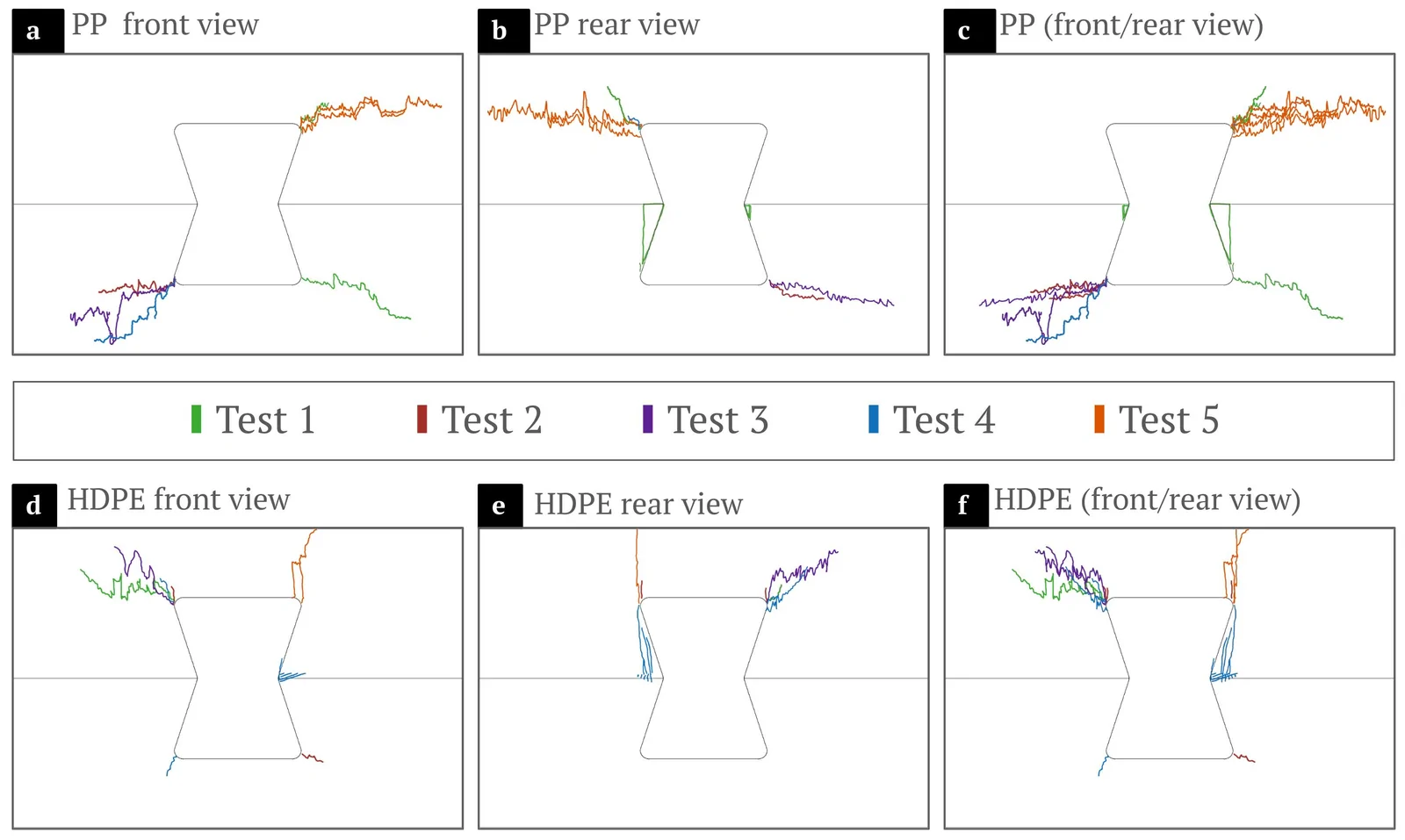
Final visible surface crack trajectories mapped from the post-test front and rear faces of the hybrid bow-tie joints: (**a**–**c**) PP specimens and (**d**–**f**) HDPE specimens. The maps represent the final visible condition and do not establish crack-initiation time, propagation sequence, or subsurface damage.

Figure 6 presents the final visible surface crack trajectories mapped on the front and rear faces of the PP and HDPE specimens. The maps show that the visible cracks are repeatedly located in the plywood adjacent to the connector region. The exact surface trajectory varies among specimens, but no visible surface crack is observed to traverse either thermoplastic insert. Internal damage within the connectors was not assessed. Because the maps were constructed from the final specimen condition, they do not establish the location or time of crack initiation, the temporal order in which individual cracks developed, or the complete three-dimensional crack path through the plywood thickness. Accordingly, the evidence supports conclusions concerning final visible crack localization rather than the temporal sequence of crack propagation.

**Figure 7:**
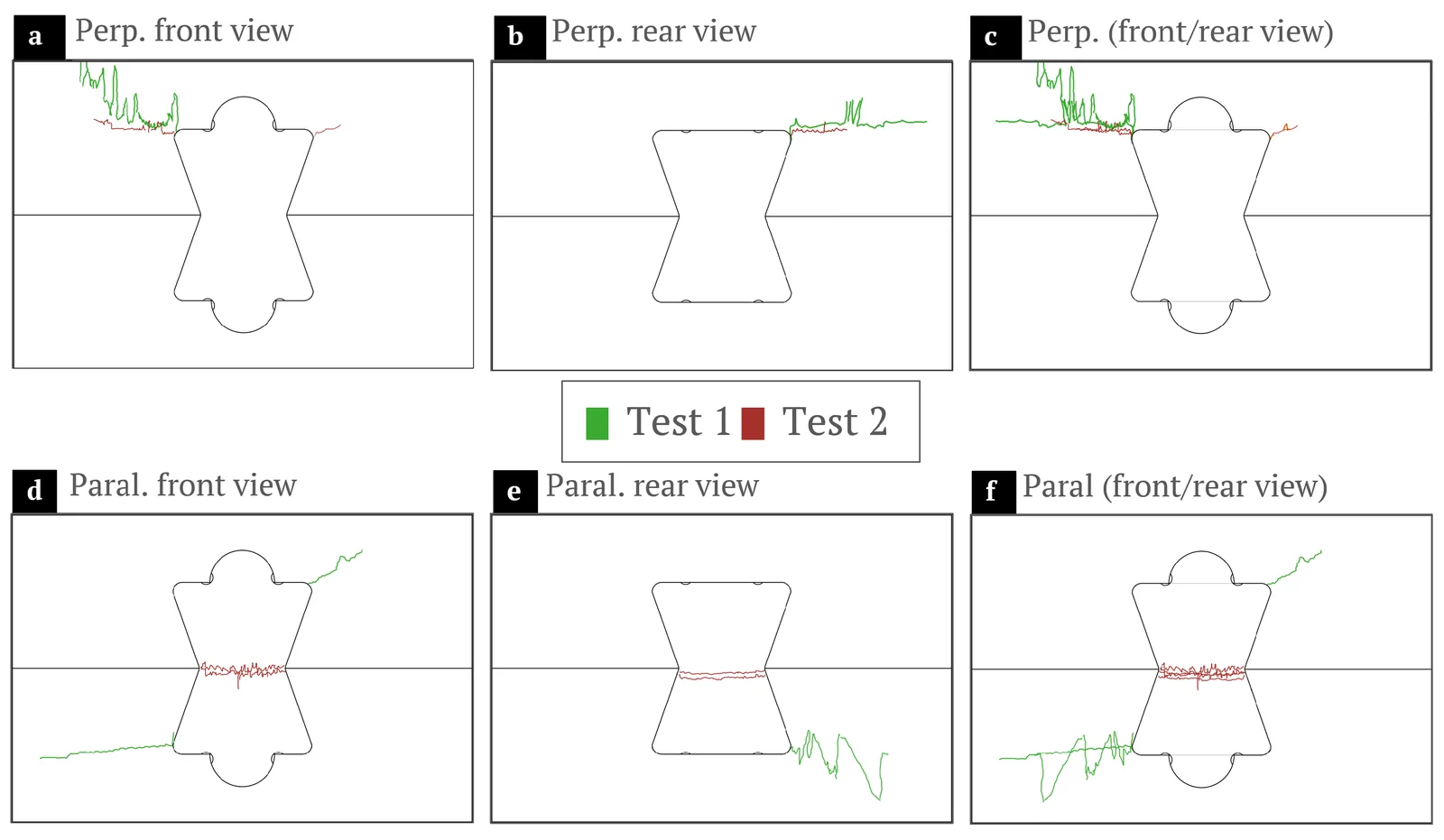
Final visible surface crack trajectories previously reported for all-plywood Skylark–WikiHouse bow-tie joints [20]: (**a**–**c**) perpendicular configuration, STD_(⊥)_; and (**d**–**f**) parallel configuration, STD_(‖)_. The historical results are included as contextual benchmarks.

For contextual comparison, Figure 7 presents the final visible crack trajectories previously reported for all-plywood Skylark–WikiHouse joints [20]. In those configurations, visible cracking occurred through both the plywood connector and the surrounding panel. The present hybrid specimens show a different final visible damage location: cracking is observed in the plywood panels, whereas the PP and HDPE inserts remain unruptured.

This comparison indicates that thermoplastic substitution changes the final visible rupture location from the plywood connector to the surrounding plywood panels. It does not demonstrate that the thermoplastic connectors retained their initial stiffness, dimensional accuracy, load-carrying capacity, or suitability for reuse after testing. The comparison is also descriptive because the all-plywood specimens belong to a separate experimental campaign.

The present observations therefore support an interpretation of final visible damage localization rather than an experimentally demonstrated crack-arrest mechanism. In all ten hybrid specimens, visible cracking was confined to the surrounding plywood, while neither thermoplastic connector exhibited complete rupture before the applicable test-termination criterion. This final damage state is consistent with the intended suture-inspired design concept, in which an interlocking compliant interface is introduced to modify load transfer and deformation. However, the present post-test observations do not establish whether cracks were initiated, redirected, slowed, or arrested by the thermoplastic connector. In particular, the absence of continuous local strain or crack measurements prevents determination of the temporal sequence of damage development. The biomimetic interpretation should therefore be regarded as a design-level analogy supported by the observed final damage localization, rather than as direct experimental evidence of crack arrest.

### 3.4. Biomimetic Interpretation and Engineering Implications

The bow-tie geometry transfers tensile load between the thermoplastic insert and the plywood panels through direct contact along the interlocking surfaces, providing a structural analog of the interlocking geometry found in natural sutures. The measured differences between PP and HDPE indicate that connector-material substitution is associated with changes in the global stiffness–deformation response while maintaining similar mean maximum tensile load. The lower global stiffness, greater crosshead displacement at maximum load, and greater external mechanical work measured for the HDPE group are consistent with a more compliant global joint response.

The greater nominal stiffness of PP is consistent with its steeper ascending force–displacement response and lower mean crosshead displacement at maximum load. However, the present experiments cannot determine the extent to which these differences arise from bulk connector compliance, the properties of the molded components, interfacial contact and friction, local geometric fit, or other sources of specimen and system compliance. Accordingly, the PP–HDPE comparison should be interpreted as a joint-level experimental contrast rather than as a direct measurement of the constitutive behavior of the two thermoplastic materials.

Although the calculated stiffness reflects the global response of the specimen–grip–machine system rather than isolated material deformation, the comparison between the PP and HDPE groups provides evidence that connector-material substitution is associated with substantial differences in global joint response under the controlled experimental configuration. Because the crosshead displacement includes contributions from connector deformation, plywood deformation, relative movement at the contact surfaces, grip compliance, and testing-machine compliance, the present measurements do not permit these contributions to be partitioned independently. Accordingly, the observed 33% reduction in global stiffness and 63% increase in displacement should be interpreted as changes in the global joint response associated with PP-to-HDPE substitution, rather than as quantities attributable exclusively to deformation of the thermoplastic connector.

Similarly, the greater value of $W_{\mathrm{pre}}$ for HDPE reflects the greater external work applied to the complete specimen–grip–machine system before maximum load. It should not be interpreted as direct evidence of greater energy dissipation exclusively by the HDPE connector. The measured response is consistent with greater global deformation capacity of the HDPE-based joint, while the local mechanisms contributing to this response remain unresolved.

The macroscopic results therefore support the use of connector material as a design variable for modifying the global stiffness–deformation response of digitally fabricated timber joints. They do not, however, directly demonstrate local stress redistribution, crack arrest, or a unique micromechanical load-transfer mechanism. Quantifying local contact pressure, friction, strain localization, crack-initiation timing, and three-dimensional damage evolution would require additional measurements or computational analysis, such as full-field optical methods, interrupted testing, acoustic monitoring, or contact-based finite-element modeling.

Within the investigated configuration, connector-material selection provides a means of modifying global joint stiffness, displacement at maximum load, and external mechanical work while retaining the exact same biomimetic bow-tie geometry. PP produces the stiffer global response, whereas HDPE is associated with greater pre-peak crosshead deformation and external work. The similar mean maximum tensile loads, approximately 6.6 kN for both groups, indicate that the principal experimental contrast in the present dataset lies in the global stiffness–deformation response rather than the mean maximum load. By treating connector-material selection as a structural design variable, material substitution can complement geometric design when tailoring the global response of digitally fabricated timber joints. This interpretation remains specific to the investigated joint geometry, materials, manufacturing procedures, loading protocol, and measurement configuration.

## 4. Conclusions

This study evaluated the bio-inspired structural performance and failure localization of Skylark–WikiHouse hybrid bow-tie joints incorporating compression-molded polypropylene (PP) and high-density polyethylene (HDPE) connectors. The bio-inspired joint geometry was used as a design framework for investigating whether connector-material selection can modify the global mechanical response while retaining the same interlocking geometry. The experimental results support the following conclusions:

1. The PP and HDPE joints reached mean maximum tensile loads of 6.61 and 6.65 kN, respectively, corresponding to a difference below 1%. Thus, within the investigated configuration and experimental dataset, the principal difference between the two connector materials was observed in the global deformation response rather than the mean maximum load.
2. Relative to PP, the HDPE joints exhibited 33% lower mean global stiffness, 63% greater mean crosshead displacement at maximum load, and 70% greater mean external mechanical work up to maximum load. HDPE consequently produced a more compliant response, whereas PP provided greater global stiffness and reached maximum load at a lower mean displacement. Because these quantities were obtained from force and testing-machine crosshead displacement, they characterize the global specimen–grip–machine response and should not be interpreted as direct measurements of intrinsic connector stiffness, local connector deformation, or connector-specific energy dissipation.
3. Final visible cracking was located in the surrounding plywood in all ten hybrid specimens, while no thermoplastic connector ruptured before the applicable test-termination criterion was reached. The HDPE connectors nevertheless exhibited greater visible elongation and localized narrowing than the PP connectors. These observations establish a repeatable final visible damage localization in the tested specimens but do not establish the temporal sequence of crack initiation and propagation or demonstrate a crack-arrest mechanism.
4. The measured differences between PP and HDPE identify connector material as a practical design variable for tailoring the global stiffness–deformation response of digitally fabricated timber joints while retaining a fixed bow-tie geometry. The observed contrast is compatible with the intended suture-inspired design concept, but the present experiments do not independently resolve the contributions of molded-component properties, interfacial contact and friction, geometric fit, or system compliance.

Overall, both thermoplastic connector groups reached mean maximum tensile loads of approximately 6.6 kN while exhibiting substantial differences in global joint stiffness, crosshead displacement at maximum load, and external mechanical work. PP exhibited the higher global stiffness, whereas HDPE exhibited greater crosshead displacement at maximum load and external mechanical work. These findings support connector-material selection as a complementary design variable for tuning the global response of digitally fabricated timber connections without changing the bow-tie geometric profile. The biomimetic interpretation is restricted to the design level: the final visible damage localization in the surrounding plywood is consistent with the intended suture-inspired interlocking concept, but the present post-test observations do not provide direct experimental evidence of crack arrest or local stress redistribution. Future research should characterize the mechanical properties of the molded connectors directly and investigate local deformation fields, interfacial contact and friction, and the temporal evolution of damage using appropriate experimental or validated computational methods.

## Figures and Tables

**Table 1:** Identification and nominal reference properties of the constituent materials. The plywood properties were obtained from a previous orthotropic characterization of the same plywood batch used in the present study [49]. The PP and HDPE properties were obtained from the corresponding manufacturer datasheets and were not measured directly from the compression-molded connectors.

Property	Radiata Pine Plywood	PP	HDPE
Material identification	Structural plywood, seven plies	R520Y	2520
Density (g/cm^3^)	0.48–0.55	0.90	0.95–0.96
Moisture content (%)	10.5±0.5	–	–
Tensile strength (MPa)	$T_{x}=28.40$; $T_{y}=29.66$	31	27–29
Tensile modulus (GPa)	$E_{x}=5.83$; $E_{y}=4.68$	1.37	0.95–1.05
Elongation at break (%)	–	>500	>500

**Table 2:** Specimen-level maximum tensile load and crosshead displacement at maximum load for the PP and HDPE joints.

PP Joints			HDPE Joints		
Specimen	$F_{\max}$(kN)	$\delta_{\max}$(mm)	Specimen	$F_{\max}$(kN)	$\delta_{\max}$(mm)
P1	6.02	11.11	P6	7.10	14.04
P2	6.75	7.69	P7	6.56	11.56
P3	5.79	9.21	P8	6.04	12.09
P4	7.49	10.56	P9	7.16	22.52
P5	6.97	8.01	P10	6.38	15.56
Mean	6.61	9.31	Mean	6.65	15.16
Standard deviation	0.70	1.51	Standard deviation	0.48	4.41
Coefficient of variation (%)	10.55	16.18	Coefficient of variation (%)	7.21	29.13

**Table 3:** Summary of the global tensile-response parameters for the PP and HDPE joints and the previously reported all-plywood configurations. Values are reported as mean ± standard deviation. The all-plywood values are contextual benchmarks from a separate experimental campaign [20].

Connector Configuration	*n*	$F_{\max}$ (kN)	$\delta_{\max}$ (mm)	$k_{10-40}$ (N/mm)	$W_{\mathrm{pre}}$ (J)
PP	5	6.61±0.70	9.31±1.51	875.9±79.3	35.2±7.6
HDPE	5	6.65±0.48	15.16±4.41	587.4±81.8	59.7±22.7
All-plywood STD_(‖)_	2	7.10±0.38	8.23±0.63	1203.0±9.2	32.0±0.7
All-plywood STD_(⊥)_	2	7.05±1.19	9.04±2.05	1144.6±13.3	34.3±17.9

## Data Availability

The original contributions presented in this study are included in the article. Further inquiries can be directed to the corresponding authors.
